# Treatment With Resveratrol Ameliorates Mitochondrial Dysfunction During the Acute Phase of Status Epilepticus in Immature Rats

**DOI:** 10.3389/fnins.2021.634378

**Published:** 2021-03-05

**Authors:** Jaroslava Folbergrová, Pavel Ješina, Jakub Otáhal

**Affiliations:** Institute of Physiology of the Czech Academy of Sciences, Prague, Czechia

**Keywords:** reactive oxygen species, immature rats, status epilepticus, superoxide anion production, deficiency of mitochondrial complex I activity, resveratrol, protection

## Abstract

The aim of the present study was to elucidate the effect of resveratrol (natural polyphenol) on seizure activity, production of ROS, brain damage and mitochondrial function in the early phase of status epilepticus (SE), induced in immature 12 day-old rats by substances of a different mechanism of action (Li-pilocarpine, DL-homocysteic acid, 4-amino pyridine, and kainate). Seizure activity, production of superoxide anion, brain damage and mitochondrial function were assessed by EEG recordings, hydroethidium method, FluoroJadeB staining and Complex I activity measurement. A marked decrease of complex I activity associated with the acute phase of SE in immature brain was significantly attenuated by resveratrol, given i.p. in two or three doses (25 mg/kg each), 30 min before, 30 or 30 and 60 min after the induction of SE. Increased O_2_^.–^ production was completely normalized, brain damage partially attenuated. Since resveratrol did not influence seizure activity itself (latency, intensity, frequency), the mechanism of protection is likely due to its antioxidative properties. The findings have a clinical relevance, suggesting that clinically available substances with antioxidant properties might provide a high benefit as an add-on therapy during the acute phase of SE, influencing also mechanisms involved in the development of epilepsy.

## Introduction

Existing data clearly indicate that seizures and status epilepticus (SE) are associated with oxidative stress (Patel, 2004; Waldbaum and Patel, 2010; Shin et al., 2011; Folbergrová and Kunz, 2012; Rowley and Patel, 2013; Williams et al., 2015; Folbergrová, 2016). Our recent studies have demonstrated that oxidative stress (demonstrated by the increased production of O_2_^.–^ in various brain regions) (Folbergrová et al., 2012, 2016) and by the elevation of mitochondrial oxidative damage markers, 3-nitrotyrosine, 4-hydroxynonenal and protein carbonyls) (Folbergrová et al., 2010) and mitochondrial dysfunction (particularly a marked inhibition of respiratory chain complex I activity (Folbergrová et al., 2007, 2010, 2016) occur also in immature brain and may thus be considered a general phenomenon (Folbergrová and Kunz, 2012; Folbergrová, 2013). Recent studies suggest that targeting oxidative stress can ameliorate alterations associated with the acute phase of SE and improve also disease outcome (e.g., Pauletti et al., 2017).

Many efforts have been aimed at developing the substances capable of detoxifying reactive oxygen and nitrogen species (ROS and RNS) and their damaging effects (Linseman, 2009; Batinic-Haberle et al., 2010). Synthetic metalloporphyrin catalytic antioxidants (small molecule mimics of SOD and/or catalase) have appeared as a novel neuroprotective agents (Patel and Day, 1999; Reboucas et al., 2008; Sheng et al., 2014). Oxidative stress and neuronal damage associated with status epilepticus in adult animals could be attenuated by some of these compounds (Rong et al., 1999; Liang et al., 2012). We have shown that both superoxide anion formation and the deficiency of complex I activity associated with SE in immature rats could be prevented or substantially attenuated with SOD mimic Mn (III) tetrakis (1-methyl-4-pyridyl) porphyrine pentachloride (MnTMPYP, from Calbiochem), a nitroxide antioxidant and the superoxide dismutase mimic 4-hydroxy-2,2,6,6-tetramethylpiperidine-1-oxyl (Tempol, from Sigma) and by a peroxynitrite scavenger and decomposition catalyst 5,10,15,20-tetrakis (4-sulfonatophenyl) porphyrinate Iron (III) (FeTPPS from Calbiochem) (Folbergrová et al., 2007, 2010, 2011, 2016; Folbergrová and Kunz, 2012). In addition, treatment with these antioxidants resulted in a partial amelioration of neuronal degeneration associated with SE in immature rats (Folbergrová et al., 2011, 2016).

It is well-recognized, that acquired epilepsy develops in otherwise healthy brain after the initial “epileptogenic insult” (such as status epilepticus, hypoxic-ischemic insults, infection, trauma, stroke etc.). It triggers vast cascade of multilevel processes which in some individuals finally result in occurrence of spontaneous recurrent seizures, i.e., epilepsy. Epileptogenic insult, epileptogenesis and epilepsy most likely represent independent pharmacological targets. Recently we have observed protective effect of a natural polyphenolic compound present in red wine, resveratrol (RSV) (3,5,4’-tri-hydroxy-trans-stilbene), during epileptogenesis, i.e., long period, up to 4 weeks, following the epileptogenic insult, namely Li-Pilocarpine status epilepticus in immature rats (Folbergrová et al., 2018).

The aim of the present study was to discover effect of resveratrol on seizure activity, production of ROS, neuronal damage and mitochondrial function in the early phase of epileptogenic insult itself, i.e., status epilepticus induced in immature rats. For induction of status epilepticus we have utilized 4 substances of a different mechanism of action, namely DL-homocysteic acid, 4-aminopyridine, Li-pilocarpine and kainate, offering the possibility for general conclusions on resveratrol effect during epileptogenic insult.

## Materials and Methods

### Animals

Immature 12-day-old male Wistar rats were used for these experiments. Twelve-day-old rats were chosen because of the level of brain maturation which is comparable to the early postnatal period in human infants (Dobbing, 1970). The rat pups were removed from their dams 1 h before the experiment. They were kept in plastic observation chambers on an electrically heated pad at 34°C (i.e., the temperature of the nest), with the exception of surgery. The protocol of experiments was approved by the Animal Care and Use Committee of the Institute of Physiology, Academy of Sciences of the Czech Republic, in agreement with Animal Protection Law of the Czech Republic, which is fully compatible with the guidelines of the European Community Council directives 86/609/EEC. The Institute possesses The Statement of Compliance with Standards of Humane Care and Use of Laboratory Animals #A5228-01 from NIH. All efforts were made to minimize animal suffering and to reduce the number of animals used.

### Surgery

The animals were anesthetized with isoflurane and fixed in a stereotaxic apparatus, modified for rat pups (Folbergrová et al., 2000). For DL-HCA and 4-AP application, bilateral stainless steel guide cannulae (26-gauge, 4 mm in length, Plastics One, Germany) were stereotaxically implanted 1 mm above the lateral ventricles (AP:0.7 mm caudal from the bregma; L: ± 1.5 mm; V: 3.3 mm from the skull surface). Cannulae were fixed to the skull with dental acrylic. After the surgery animals were returned to their mothers in home cages to recover.

### EEG Recordings and Analysis

After surgery, animals recovered 2 h and then were connected to the EEG system (Pentusa, TDT, United States) and continual EEG were recorded on 1 kHz and stored for offline analysis. The EEG recording covered baseline (20 min) and whole period of SE development up to 90 min duration. Offline analyses were performed in Spike2 (CED, United Kingdom) and Matlab (Mathworks, United States) software. Epileptic spikes were detected as suprathreshold events and quantified in 60 s long bins.

### Seizure Induction

DL-HCA (from Aldrich, Germany) and 4-AP (SigmaAldrich) were dissolved in sterile saline and the pH adjusted to ∼7.0, only freshly prepared solutions were used. Bilateral i.c.v. infusions of DL-HCA (600 nmol/side), 4-AP (100 nmol/side) or saline were made in a volume of 0.5 μl at a rate of 0.17 μl/min using a SP200i infusion pump (WPI, United States) through stainless steel internal cannulae (33 gauge, 5 mm in length, Plastics One, Germany), each connected by a polyethylene tube to a 10 μl Hamilton syringe. To induce Li-Pilo SE, LiCl (SigmaAldrich) was dissolved in redistilled water and administered i.p. to PD11 immature rats (127 mg/kg). After 24 h, pilocarpine (SigmaAldrich), dissolved in redistilled water was given i.p. (35 mg/kg) to lithium pretreated pups. To induce kainate SE, KA (Tocris Bioscience, Bristol, United Kingdom) was dissolved in saline and given i.p. (6 mg/kg). Control animals received corresponding volumes of the appropriate vehicles.

### The Effect of Resveratrol

**FIGURE 1:**
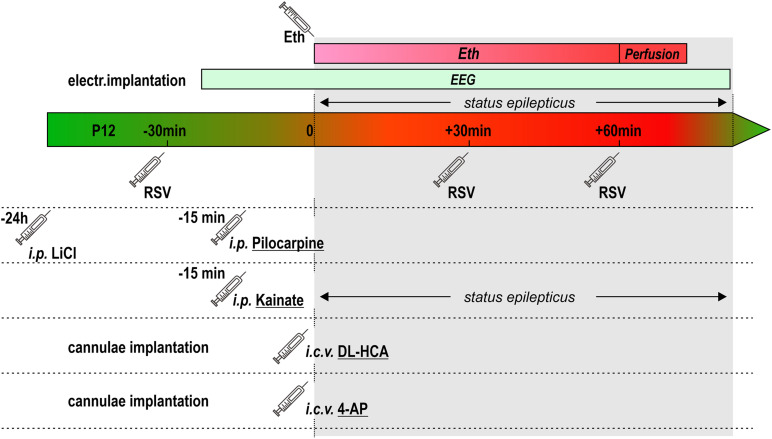
Schema of experimental design.

For assessing a potential protective effect of a natural polyphenolic compound resveratrol (RSV), RSV (from Sigma Co.) was dissolved in DMSO and then diluted with PBS (final concentration of DMSO ∼5%). Only freshly prepared solutions, kept in dark, were used for applications. Resveratrol was given i.p. in two or three doses (25 mg/kg each), 30 min before, 30 or 30 and 60 min after induction of SE. The schema of experimental design of the current study can be seen in Figure 1.

### Superoxide Anion Determination

Production of superoxide anion (O_2_^.–^) in different brain regions *in situ* was determined using hydroethidium (Het) method (Bindokas et al., 1996), adopted for immature rats, as described in detail in our previous work (Folbergrová et al., 2012). Het was given by i.p. injection immediately before infusion of DL-HCA or 4-AP and ∼15 min after i.p. administration of Li-Pilo or KA (final concentration 10 mg/kg). Sixty minutes after the application of Het, rat pups were deeply anesthetized with 20% (w/v) urethane and transcardially perfused with 0.01 M phosphate buffered saline (PBS), pH 7.4, followed by a fixative solution [4% (w/v)] paraformaldehyde in 0.1 M phosphate buffer, pH 7.4. The brains were removed from the skull, postfixed for 3 h at 4°C in the same fixative, then cryoprotected in sucrose of increasing concentrations (10, 20, and 30% (w/v), respectively) in 0.1 M phosphate buffer, pH 7.4 and finally frozen in dry ice. Coronal sections (50 μm) were cut through the brain in a cryostat and mounted onto the gelatinated slides. All procedures were performed under the reduced light.

The level of the oxidized products of Het was assessed microscopically by detection of their fluorescence (>600 nm). Pictures of the selected regions of interest (hippocampal fields CA1, CA3, and DG, primary somatosensory cortex and dorsal thalamus) of the same size and orientation, were captured (AP −3.5 to −4.0 according to Paxinos and Watson (Paxinos and Watson, 1998), with cooled camera mounted onto upright microscope (10 x magnification lens). Camera settings remained unchanged throughout the evaluation of the current set of tissue sections of animals from one experimental day, treated with the same solution of Het. The group comprised always at least three saline-treated controls, three animals with convulsant drug alone and three with convulsant drug plus resveratrol. Fluorescence signal (represented as integral intensity of the given region) was normalized by values of the control animals of the current set. Results are expressed as percentage of saline-treated animals.

### Brain Damage Analysis

Brain damage was evaluated in Li-Pilo model of SE. At 24 h following SE, rat pups from Li-Pilocarpine (*n* = 9) and Li-Pilocarpine + resveratrol (*n* = 8) groups were subjected to fixation procedure (see its detailed description in the section “Superoxide Anion Determination”). Coronal 50 μm thin slices were cut and stained with Fluoro-Jade B (Histochem, United States) as previously used and described in details by our group (Folbergrová et al., 2012, 2016). To assess neurodegeneration and potential protective effect of resveratrol, we have performed semiquantitative grading (using a score) of number of Fluoro-Jade B positive cell in regions of interest (ROI), spatially corresponding to regions evaluated by ethidium method. Neurodegeneration was assessed in hippocampal regions CA1, CA3 and dentate gyrus (DG), sensorimotor cortex (Cx), and mediodorsal thalamic nuclei (Thal). Position of ROIs, selected consistently through all animals, are illustrated in Figure 4B. A semiquantitative scale was used to assess the brain damage; score 0: <7 neurons, score 1: 7–15 neurons, score 2: 16–25 neurons, score 3: 26–40 neurons, score 4: >40 neurons.

### Isolation of Mitochondria

Mitochondrial fractions were isolated according to Liang et al. (Liang et al., 2000), as described in detail in our previous works (Folbergrová et al., 2007, 2016). All procedures were performed at 4°C. Cerebral cortices (weighing ∼250 mg) were used for each mitochondrial preparation. 10% (w/v) homogenates in ice-cold isolation buffer (70 mM sucrose, 210 mM mannitol, 5 mM Tris-HCl, 1 mm EDTA, pH 7.4) were prepared with Elvehjem-Potter type glass-Teflon homogenizers manually by twenty slow up-and-down strokes. Homogenates were centrifuged at 600 × g for 5 min at 4°C, the postnuclear supernatant was centrifuged at 17,000 × g for 10 min at 4°C. Mitochondrial pellet was resuspended with 100 μl 50 mM Tris-HCl (pH 7.4). Fresh isolated mitochondria were used for protein determination. Aliquots of mitochondria frozen in liquid nitrogen and stored at −70°C were used for determinations of complex I and citrate synthase activities (performed within 1 week).

### Enzyme Assays

Activities of mitochondrial respiratory chain complex I and citrate synthase were measured at 30°C in a total reaction volume of 1 ml using Shimadzu 1601 spectrophotometer. Duplicate determinations were carried out with each mitochondrial sample.

#### Complex I

NADH-ubiquinone oxidoreductase (EC 1.6.5.3) activity was determined as the rotenone-sensitive rate of NADH oxidation at 340 nm. The reaction mixture contained: 25 mM potassium phosphate (pH 7.2), 10 mM MgCl_2_, 1 mM KCN, 0.25% fatty acid-free bovine serum albumin (BSA), 0.1 mM NADH and approximately 50 μg of mitochondrial protein. The reaction was initiated by the addition of CoQ10 (decylubiquinone, final concentration 50 μM). After 2 min, 2 μl of rotenone were added (final concentration 5 μM) and the inhibited rate was followed for further 2 min (Folbergrová et al., 2007, 2016).

#### Citrate Synthase

Citrate synthase (EC 4.1.3.7) activity was determined as the rate of color change of 5,5’-dithiobis-(2-nitrobenzoic) acid (DTNB) at 412 nm. The reaction mixture contained 100 mM Tris-HCl (pH 8.1), 0.2 mM DTNB, 0.1% Triton X-100, 0.1 mM acetyl-CoA and ∼20 μg of mitochondrial protein. The reaction was initiated by the addition of 20 μl of 10 mM oxaloacetate (final concentration 0.2 mM) (Folbergrová et al., 2007, 2016).

Activity of complex I was expressed as nmol/min/mg protein. To correct for the potential variations in mitochondrial contents in the samples, activities can also be expressed as a ratio to citrate synthase.

#### Protein Determination

Mitochondrial protein concentration was estimated by Bradford’s method, with bovine serum albumin as a standard.

### Statistics

Statistical analysis was performed in SigmaPlot 13 software (Systat Software Inc., United States). The data were evaluated by one-way ANOVA with Newmann-Keul’s *post hoc* test or by *t*-test where appropriate. The level of statistical significance was set to 5%.

## Results

### The Behavioral Pattern of Seizures

All four convulsants induced SE that was characterized by generalized clonic-tonic seizures in DL-HCA and 4-AP models and by generalized clonic seizures in Li-Pilo and KA model, in the latter case accompanied by mild tonic extensions. Detailed description is given in our previous studies (Folbergrová et al., 2000, 2016).

### Effect of Resveratrol on Behavioral Pattern and on Electrographic Activity in Li-Pilo SE

**FIGURE 2:**
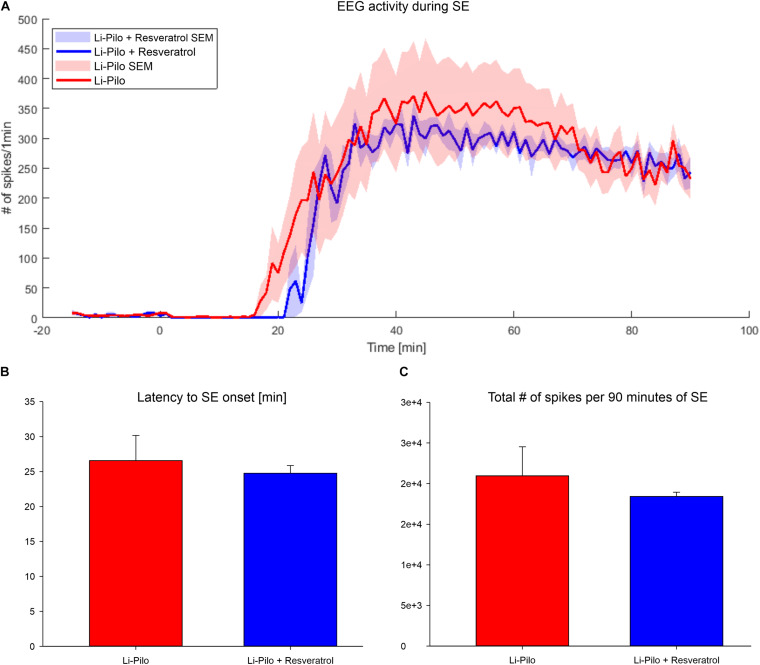
**(A)** EEG activity during Li-Pilo status epilepticus has been determined as number of epileptic spikes in 60 s bins. Time zero is the time of pilocarpine application. Red line, convulsant agent alone; Blue line, convulsant agent plus resveratrol. **(B)** Latency to the first electrographic seizure does not differ after resveratrol treatment (*P* = 0.753). Red column, convulsant agent alone; Blue column, convulsant agent plus resveratrol. **(C)** Total number of detected epileptic spikes during 90 min of SE duration was not influenced by resveratrol suggesting no anticonvulsant effect (*P* = 0.668). Red column, convulsant agent alone; Blue column, convulsant agent plus resveratrol.

In all four models studied, latency to the first behavioral seizure and character of SE were not influenced by resveratrol. Effect of resveratrol on electrographic pattern has been analyzed in Li-Pilo model. Latency to the first electrographic seizure and severity of SE, as assessed by number of spikes during first 90 min, were not influenced by resveratrol treatment (Figure 2).

### Generation of Superoxide Anion During Seizures

**FIGURE 3:**
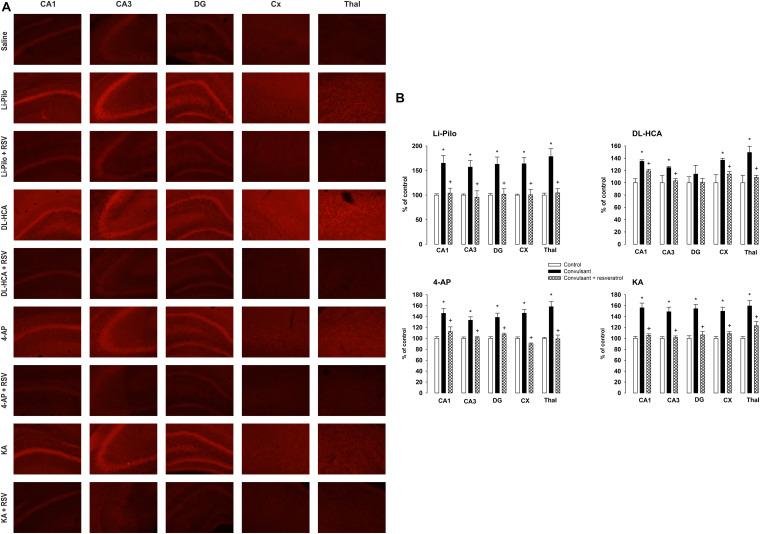
**(A)** Fluorescence of the oxidized products of hydroethidium (reflecting superoxide anion production), assessed microscopically by fluorescence (>600 nm), in various brain structures following 60 min lasting SE induced by Li-Pilocarpine (Li-Pilo), homocysteic acid (DL-HCA), 4-aminopyridine (4-AP) or kainic acid (KA). Upper image for each model, convulsant agent alone; lower image for each model, convulsant agent plus resveratrol. **(B)** Effect of resveratrol on superoxide anion formation at 60 min following the onset of SE, induced in immature rats by Li-Pilo, DL-HCA, 4-AP, or KA. White columns, saline-treated controls; black columns, convulsant agent alone; cross-hatched columns, convulsant agent plus resveratrol. Results are expressed in percent, compared to 100% in the control animals. Mean values for 5–6 animals ± SEM. *P < 0.05 as compared with saline; ^†^*P* < 0.05 as compared with convulsant agent alone. CA1 and CA3, hippocampal fields; DG, dentate gyrus; CX, cerebral cortex; Thal, dorsal thalamus.

As can be seen in Figures 3A,B, fluorescent signal of the oxidized products of Het (reflecting O_2_^.–^ production) significantly increased in all the studied brain structures, namely CA1, CA3, and DG of hippocampus, cerebral cortex and thalamus of immature rats after SE lasting 60 min in all four models, with the exception of DG in DL-HCA model.

**FIGURE 4:**
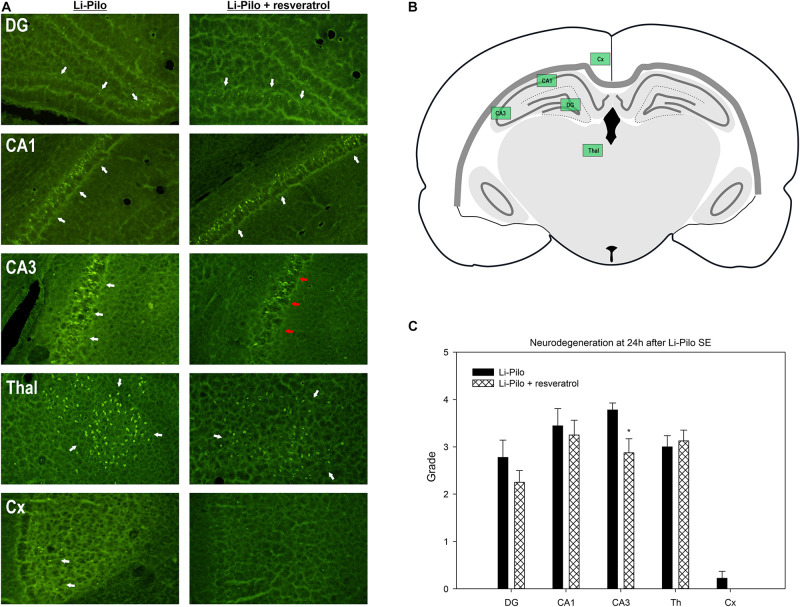
Effect of resveratrol on brain damage. **(A)** Fluoro-Jade B staining revealed neurodegeneration in the explored regions (white arrows). Resveratrol treatment provided neuroprotective effect in CA3 region (red arrows). **(B)** Schema of investigated regions. **(C)** Brain damage evaluated by semiquantitative grading of FJB positive cells (description of score employed is given in the section “Materials and Methods”). *P < 0.05 as compared with Li-Pilo alone.

### Effect of Resveratrol on O_2_^.–^ Production

Figure 3B (cross-hatched columns) demonstrates that RSV provided a complete protection in Li-Pilo, 4-AP and KA models and significantly reduced the fluorescence signal during SE induced by DL-HCA (see also Figure 3A, lower row of images).

### Effect of Resveratrol on Brain Damage

Status epilepticus induced by Li-Pilocarpine resulted in a perceptible neuronal damage as revealed by Fluoro-Jade B staining at 24 h after SE. Structures of hippocampal formation, namely CA1 and CA3, as well as dentate gyrus (DG) and mediodorsal thalamic nuclei have been bilaterally affected and various number of Fluoro-Jade B positive cells has been identified under both control (Li-Pilo only) and resveratrol treated conditions. Sensorimotor cortex, however, revealed only a minimal or none neuronal damage. Semiquantitative evaluation of neuronal damage in selected regions of interest revealed partial but still significant neuroprotection in CA3 field of hippocampus (3.8 ± 0.15 vs. 2.9 ± 0.3, *P* = 0.023) while other evaluated regions did not show signs of neuroprotection (Figures 4A,C).

### Effect of Resveratrol on Inhibition of Complex I Activity

**FIGURE 5:**
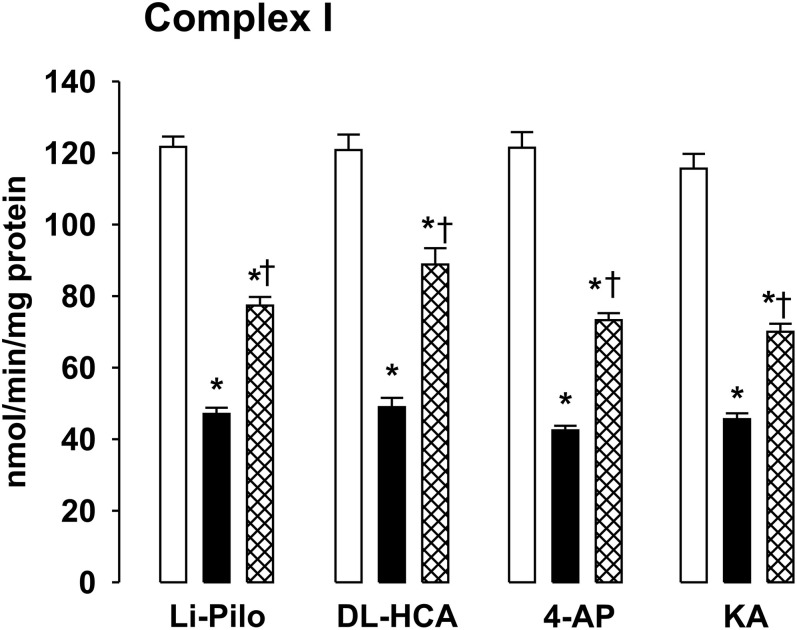
Effect of resveratrol on mitochondrial complex I activity at ∼20 h of survival after SE induced in immature rats by Li-Pilo, DL-HCA, 4-AP, or KA. White columns, controls; black columns, convulsant agent alone; cross-hatched columns, convulsant agent plus resveratrol. Results are expressed as nmol/min/mg protein. Mean values for 4–6 animals ± SEM. *P < 0.05 as compared with controls; ^†^*P* < 0.05 as compared with convulsant agent alone.

Our previous studies have demonstrated that SE induced in immature rats by DL-HCA (Folbergrová et al., 2007) or by 4-AP, Li-Pilo, and KA (Folbergrová et al., 2016) leads to a marked deficiency of complex I activity, corresponding to more than 50%. As evident in Figure 5, the inhibition of complex I activity was in all four models studied significantly attenuated by the treatment with RSV. Although significant, the protection was only partial since the activities of complex I after treatment with RSV remained significantly lower as compared with the appropriate controls. The same decrease of complex I activity and the same extent of protection provided by resveratrol was evident when the activity was expressed both as the specific activity (Figure 5) and as a ratio to citrate synthase (data not shown).

## Discussion

The important finding of the present study is the proof that resveratrol, naturally occurring polyphenolic compound, was able significantly reduce mitochondrial dysfunction associated with the acute phase of SE in immature rats.

The crucial question arises whether the protective effect of RSV could not be due to an anticonvulsant effect. However, behavioral pattern of seizures (severity, frequencies, duration) observed in all the studied models did not differ between groups with convulsant agent and resveratrol and those with convulsant compound alone. In addition, lack of an anticonvulsant property of RSV was confirmed in Li-Pilo model by EEG recordings. Our findings are thus in agreement with recent reports of Tomaciello et al. (2016). These authors employing three different seizure models in adult mice did not observe an obvious anticonvulsant effect of RSV, only a trend toward a delay in seizure latency. Thus, our findings are compatible with the statement that the protective effect of RSV is most likely due to its antioxidant properties.

Neuroprotective effect of RSV has been observed in various models of neurological disorders in adult animals (Gupta et al., 2002; Wu et al., 2009; Kroon et al., 2010; Sakata et al., 2010; Shetty, 2011; Wang et al., 2013; Mishra et al., 2015; Castro et al., 2017). Its employment has some advantages, since RSV enters the brain after a peripheral administration and it does not seem to have adverse effects (Shetty, 2011). It seems likely that substances interacting with multiple targets can achieve a better effect than single target therapies. Thus, RSV besides the direct antioxidant effect (Holthoff et al., 2010; Folbergrová et al., 2015) has multiple cellular effects, interfering with several signaling pathways (Kroon et al., 2010; Sahebkar, 2010; Shetty, 2011; Castro et al., 2017). Recently, it has been reported that RSV is able to activate Nrf2 (nuclear factor erythroid 2-related factor 2) which is an essential transcription factor regulating the expression of numerous endogenous antioxidant and anti-inflammatory genes and plays a crucial role in cellular defense against oxidative stress (Kesherwani et al., 2013; Narayanan et al., 2015). Importantly, our recent findings indicate that neuroprotective effect comparable to that observed with RSV can be detected in immature rats during Li-pilocarpine SE after treatment with sulforaphane, established activator of Nrf2 (manuscript in preparation). Furthermore, an increased expression (Mazzuferi et al., 2013) or activation of Nrf2 (Wang et al., 2014) have been reported recently to provide a marked protection in experimental epilepsy models in adults. Nevertheless, the precise mechanism of RSV action in our study remains to be clarified by future studies.

Whatever the mechanism, RSV prevented completely the generation of O_2_^.–^ associated with the acute phase of SE. It should be mentioned that the Het assay used for the evaluation of O_2_^.–^ formation has several limitations as discussed recently (Zielonka and Kalyanaraman, 2010; Kalyanaraman et al., 2014, 2017). These mainly concern the difficulty to distinguish microscopically the fluorescent red signal belonging to 2-hydroxyethidium (a specific product of Het reaction with O_2_^.–^) and ethidium (a product of Het reaction with other ROS and/or oxidants). Importantly, our recent findings demonstrating complete prevention of the increased fluorescent signal after the treatment animals with SOD mimetic MnTMPYP, support the involvement of superoxide anion (Folbergrová et al., 2012, 2016).

Mitochondrial dysfunction, especially a deficiency of complex I activity has been demonstrated in humans with temporal lobe epilepsy (Kunz et al., 2000; Lee et al., 2008) and in several experimental models of epilepsy in adult (Kudin et al., 2002; Chuang et al., 2004; Waldbaum and Patel, 2010; Shin et al., 2011) as well as immature animals during SE (Folbergrová et al., 2007, 2010, 2016). In agreement with our previous studies, more than 50% decrease of complex I activity was observed in all four models of SE induced in immature rats (Figure 5). The question arises what may be the underlying mechanism. We showed that in DL-HCA model, the decrease of complex I activity was not associated with changes in the size of the assembled complex I or with changes in mitochondrial content of complex I (Folbergrová et al., 2010). We have thus proposed that inactivation, namely oxidative modification of complex I, may be responsible for the deficiency of complex I activity. This assumption is supported by an extreme sensitivity of this enzyme to both oxidative and nitrosative stress (Folbergrová et al., 2010 and references therein). Furthermore, the increased ROS production detected in all four models can create conditions favoring oxidative modifications of sensitive targets. Several posttranslational oxidative modifications of complex I can occur, such as carboxylation, nitration of tyrosine (and/or tryptophane) residues, S-nitrosation of some of its protein thiols etc. (Murray et al., 2003; Brown and Borutaite, 2004; Folbergrová et al., 2010; Ryan et al., 2012, 2014). Indeed, the oxidative modification (nitration or carboxylation) of only a few subunits from the total 46 was reported to result in a pronounced inhibition of complex I activity (Murray et al., 2003; Ryan et al., 2012). Potential role of other factors beside oxidative inactivation cannot, however, be excluded. Nevertheless, the involvement of oxidative modification is supported by our recent findings demonstrating that deficiency of complex I activity could be significantly attenuated by SOD mimics Tempol (Folbergrová et al., 2007) or MnTMPYP (Folbergrová et al., 2010, 2016), by a selective peroxynitrite scavenger and decomposition catalyst FeTPPS (Folbergrová et al., 2007, 2010, 2016) and, as the present findings clearly indicate, by resveratrol.

It should be kept in mind that complex I besides being a target for ROS and RNS is also the important source of their production, especially when partially inhibited (Sipos et al., 2003; Kudin et al., 2004; Kussmaul and Hirst, 2006; Fato et al., 2008; Parihar et al., 2008). It can thus be assumed that the enhanced production of ROS and/or RNS as a consequence of complex I inhibition, may lead to a potential impairment of sufficient energy production, contribute to neuronal injury and/or epileptogenesis (Perier et al., 2005; Marella et al., 2007; Castro et al., 2017).

## Conclusion

The present study clearly demonstrates that treatment with resveratrol significantly attenuates early mitochondrial dysfunction (evident as a marked preservation of complex I activity) during the acute phase of status epilepticus in immature rats. The protective effect of resveratrol was evident in all four models, induced in immature rats with substances of a different mechanism of action and, can thus represent a general phenomenon associated with SE in immature brain. Since resveratrol does not influence seizure activity itself, the mechanism of protective action is most likely due to its antioxidative properties (as documented by diminished O_2_^.–^ production). The findings have a clinical relevance suggesting that clinically available substances with antioxidant properties might provide a high benefit as an add-on therapy during the acute phase of status epilepticus interacting with mechanisms involved in development of epilepsy.

## Data Availability Statement

The raw data supporting the conclusions of this article will be made available by the authors, without undue reservation.

## Ethics Statement

The animal study was reviewed and approved by the Ethical and Use committee of the Institute of Physiology CAS, Prague.

## Author Contributions

JF: conceptualization and writing—original draft. JF and JO: formal analysis, supervision, visualization, and writing—review and editing. JO: funding acquisition and project administration. JF, PJ, and JO: investigation and methodology. All authors contributed to the article and approved the submitted version.

## Conflict of Interest

The authors declare that the research was conducted in the absence of any commercial or financial relationships that could be construed as a potential conflict of interest.

## References

[B1] Batinic-Haberle I. Reboucas J. S. Spasojevic I. (2010). Superoxide dismutase mimics: chemistry, pharmacology, and therapeutic potential. *Antioxid Redox Signal* 13 877–918. 10.1089/ars.2009.2876 20095865 PMC2935339

[B2] Bindokas V. P. Jordan J. Lee C. C. Miller R. J. (1996). Superoxide production in rat hippocampal neurons: selective imaging with hydroethidine. *J. Neurosci.* 16 1324–1336. 10.1523/jneurosci.16-04-01324.1996 8778284 PMC6578569

[B3] Brown G. C. Borutaite V. (2004). Inhibition of mitochondrial respiratory complex I by nitric oxide, peroxynitrite and S-nitrosothiols. *Biochim. Biophys. Acta* 1658 44–49. 10.1016/j.bbabio.2004.03.016 15282173

[B4] Castro O. W. Upadhya D. Kodali M. Shetty A. K. (2017). Resveratrol for easing status epilepticus induced brain injury, inflammation, epileptogenesis, and cognitive and memory dysfunction-are we there yet? *Front. Neurol.* 8:603. 10.3389/fneur.2017.00603 29180982 PMC5694141

[B5] Chuang Y. C. Chang A. Y. Lin J. W. Hsu S. P. Chan S. H. (2004). Mitochondrial dysfunction and ultrastructural damage in the hippocampus during kainic acid-induced status epilepticus in the rat. *Epilepsia* 45 1202–1209. 10.1111/j.0013-9580.2004.18204.x 15461674

[B6] Dobbing J. (1970). “Undernutrition and the developing brain,” in *Developmental Neurobiology*, eds Himwich W. A. Thomas C. C. (Hoboken, NJ: Wiley-Blackwell), 241–261.

[B7] Fato R. Bergamini C. Leoni S. Strocchi P. Lenaz G. (2008). Generation of reactive oxygen species by mitochondrial complex I: implications in neurodegeneration. *Neurochem. Res.* 33 2487–2501. 10.1007/s11064-008-9747-0 18535905

[B8] Folbergrová J. (2013). Oxidative stress in immature brain following experimentally-induced seizures. *Physiol. Res.* 62(Suppl. 1), S39–S48. 24329702 10.33549/physiolres.932613

[B9] Folbergrová J. (2016). “Free radicals, oxidative stress, and epilepsy,” in *Reactive Oxygen Species in Biology and Human Health*, ed. Shamim A. (Boca Raton: CRC Press), 147–153.

[B10] Folbergrová J. Haugvicová R. Mareš P. (2000). Behavioral and metabolic changes in immature rats during seizures induced by homocysteic acid: the protective effect of NMDA and non-NMDA receptor antagonists. *Exp. Neurol.* 161 336–345. 10.1006/exnr.1999.7264 10683299

[B11] Folbergrová J. Ješina P. Drahota Z. Lisı V. Haugvicová R. Vojtíšková A. (2007). Mitochondrial complex I inhibition in cerebral cortex of immature rats following homocysteic acid-induced seizures. *Exp. Neurol.* 204 597–609. 10.1016/j.expneurol.2006.12.010 17270175

[B12] Folbergrová J. Ješina P. Haugvicová R. Lisı V. Houštìk J. (2010). Sustained deficiency of mitochondrial complex I activity during long periods of survival after seizures induced in immature rats by homocysteic acid. *Neurochem. Int.* 56 394–403. 10.1016/j.neuint.2009.11.011 19931336

[B13] Folbergrová J. Ješina P. Kubová H. Druga R. Otáhal J. (2016). Status epilepticus in immature rats is associated with oxidative stress and mitochondrial dysfunction. *Front. Cell. Neurosci.* 10:136. 10.3339/fncel.2016.00136 PMC4881382 27303267

[B14] Folbergrová J. Ješina P. Kubová H. Otáhal J. (2015). Resveratrol attenuates oxidative stress associated with status epilepticus in immature rats. *Epilepsia* 56 118–118.

[B15] Folbergrová J. Ješina P. Kubová H. Otáhal J. (2018). Effect of resveratrol on oxidative stress and mitochondrial dysfunction in immature brain during epileptogenesis. *Mol. Neurobiol.* 55 7512–7522. 10.1007/s12035-018-0924-0 29427088

[B16] Folbergrová J. Kunz W. S. (2012). Mitochondrial dysfunction in epilepsy. *Mitochondrion* 12 35–40. 10.1016/j.mito.2011.04.004 21530687

[B17] Folbergrová J. Otáhal J. Druga R. (2011). Effect of tempol on brain superoxide anion production and neuronal injury associated with seizures in immature rats. *Epilepsia* 52 51–51.

[B18] Folbergrová J. Otáhal J. Druga R. (2012). Brain superoxide anion formation in immature rats during seizures: protection by selected compounds. *Exp. Neurol.* 233 421–429. 10.1016/j.expneurol.2011.11.009 22108622

[B19] Gupta Y. K. Briyal S. Chaudhary G. (2002). Protective effect of trans-resveratrol against kainic acid-induced seizures and oxidative stress in rats. *Pharmacol. Biochem. Behav.* 71 245–249. 10.1016/s0091-3057(01)00663-3 11812529

[B20] Holthoff J. H. Woodling K. A. Doerge D. R. Burns S. T. Hinson J. A. Mayeux P. R. (2010). Resveratrol, a dietary polyphenolic phytoalexin, is a functional scavenger of peroxynitrite. *Biochem. Pharmacol.* 80 1260–1265. 10.1016/j.bcp.2010.06.027 20599800 PMC2934873

[B21] Kalyanaraman B. Dranka B. P. Hardy M. Michalski R. Zielonka J. (2014). HPLC-based monitoring of products formed from hydroethidine-based fluorogenic probes–the ultimate approach for intra- and extracellular superoxide detection. *Biochim. Biophys. Acta* 1840 739–744. 10.1016/j.bbagen.2013.05.008 23668959 PMC3858408

[B22] Kalyanaraman B. Hardy M. Podsiadly R. Cheng G. Zielonka J. (2017). Recent developments in detection of superoxide radical anion and hydrogen peroxide: opportunities, challenges, and implications in redox signaling. *Arch. Biochem. Biophys.* 617 38–47. 10.1016/j.abb.2016.08.021 27590268 PMC5318280

[B23] Kesherwani V. Atif F. Yousuf S. Agrawal S. K. (2013). Resveratrol protects spinal cord dorsal column from hypoxic injury by activating Nrf-2. *Neuroscience* 241 80–88. 10.1016/j.neuroscience.2013.03.015 23523995

[B24] Kroon P. A. Iyer A. Chunduri P. Chan V. Brown L. (2010). The cardiovascular nutrapharmacology of resveratrol: pharmacokinetics, molecular mechanisms and therapeutic potential. *Curr. Med. Chem.* 17 2442–2455. 10.2174/092986710791556032 20491649

[B25] Kudin A. P. Bimpong-Buta N. Y. Vielhaber S. Elger C. E. Kunz W. S. (2004). Characterization of superoxide-producing sites in isolated brain mitochondria. *J. Biol. Chem.* 279 4127–4135. 10.1074/jbc.M310341200 14625276

[B26] Kudin A. P. Kudina T. A. Seyfried J. Vielhaber S. Beck H. Elger C. E. (2002). Seizure-dependent modulation of mitochondrial oxidative phosphorylation in rat hippocampus. *Eur. J. Neurosci.* 15 1105–1114. 10.1046/j.1460-9568.2002.01947.x 11982622

[B27] Kunz W. S. Kudin A. P. Vielhaber S. Blumcke I. Zuschratter W. Schramm J. (2000). Mitochondrial complex I deficiency in the epileptic focus of patients with temporal lobe epilepsy. *Ann. Neurol.* 48 766–773. 11079540

[B28] Kussmaul L. Hirst J. (2006). The mechanism of superoxide production by NADH:ubiquinone oxidoreductase (complex I) from bovine heart mitochondria. *Proc. Natl. Acad. Sci. U.S.A.* 103 7607–7612. 10.1073/pnas.0510977103 16682634 PMC1472492

[B29] Lee Y. M. Kang H. C. Lee J. S. Kim S. H. Kim E. Y. Lee S. K. (2008). Mitochondrial respiratory chain defects: underlying etiology in various epileptic conditions. *Epilepsia* 49 685–690. 10.1111/j.1528-1167.2007.01522.x 18266755

[B30] Liang L. P. Ho Y. S. Patel M. (2000). Mitochondrial superoxide production in kainate-induced hippocampal damage. *Neuroscience* 101 563–570. 11113305 10.1016/s0306-4522(00)00397-3

[B31] Liang L. P. Waldbaum S. Rowley S. Huang T. T. Day B. J. Patel M. (2012). Mitochondrial oxidative stress and epilepsy in SOD2 deficient mice: attenuation by a lipophilic metalloporphyrin. *Neurobiol. Dis.* 45 1068–1076. 10.1016/j.nbd.2011.12.025 22200564 PMC3418969

[B32] Linseman D. A. (2009). Targeting oxidative stress for neuroprotection. *Antioxid Redox Signal* 11 421–424. 10.1089/ARS.2008.2236 18715147

[B33] Marella M. Seo B. B. Matsuno-Yagi A. Yagi T. (2007). Mechanism of cell death caused by complex I defects in a rat dopaminergic cell line. *J. Biol. Chem.* 282 24146–24156. 10.1074/jbc.M701819200 17581813

[B34] Mazzuferi M. Kumar G. Van Eyll J. Danis B. Foerch P. Kaminski R. M. (2013). Nrf2 defense pathway: experimental evidence for its protective role in epilepsy. *Ann. Neurol.* 74 560–568. 10.1002/ana.23940 23686862

[B35] Mishra V. Shuai B. Kodali M. Shetty G. A. Hattiangady B. Rao X. (2015). Resveratrol treatment after status epilepticus restrains neurodegeneration and abnormal neurogenesis with suppression of oxidative stress and inflammation. *Sci. Rep.* 5:17807. 10.1038/srep17807 26639668 PMC4671086

[B36] Murray J. Taylor S. W. Zhang B. Ghosh S. S. Capaldi R. A. (2003). Oxidative damage to mitochondrial complex I due to peroxynitrite: identification of reactive tyrosines by mass spectrometry. *J. Biol. Chem.* 278 37223–37230. 10.1074/jbc.M305694200 12857734

[B37] Narayanan S. V. Dave K. R. Saul I. Perez-Pinzon M. A. (2015). Resveratrol preconditioning protects against cerebral ischemic injury via nuclear erythroid 2-Related factor 2. *Stroke* 46 1626–1632. 10.1161/STROKEAHA.115.008921 25908459 PMC4442036

[B38] Parihar M. S. Parihar A. Villamena F. A. Vaccaro P. S. Ghafourifar P. (2008). Inactivation of mitochondrial respiratory chain complex I leads mitochondrial nitric oxide synthase to become pro-oxidative. *Biochem. Biophys. Res. Commun.* 367 761–767. 10.1016/j.bbrc.2008.01.015 18191636

[B39] Patel M. (2004). Mitochondrial dysfunction and oxidative stress: cause and consequence of epileptic seizures. *Free Radic. Biol. Med.* 37 1951–1962. 10.1016/j.freeradbiomed.2004.08.021 15544915

[B40] Patel M. Day B. J. (1999). Metalloporphyrin class of therapeutic catalytic antioxidants. *Trends Pharmacol. Sci.* 20 359–364. 10.1016/s0165-6147(99)01336-x 10462758

[B41] Pauletti A. Terrone G. Shekh-Ahmad T. Salamone A. Ravizza T. Rizzi M. (2017). Targeting oxidative stress improves disease outcomes in a rat model of acquired epilepsy. *Brain* 140 1885–1899. 10.1093/brain/awx117 28575153 PMC6248577

[B42] Paxinos G. Watson C. (1998). *The Rat Brain in Stereotaxic Coordinates.* San Diego: Academic Press.

[B43] Perier C. Tieu K. Guegan C. Caspersen C. Jackson-Lewis V. Carelli V. (2005). Complex I deficiency primes Bax-dependent neuronal apoptosis through mitochondrial oxidative damage. *Proc. Natl. Acad. Sci. U.S.A.* 102 19126–19131. 10.1073/pnas.0508215102 16365298 PMC1323177

[B44] Reboucas J. S. Spasojevic I. Batinic-Haberle I. (2008). Quality of potent Mn porphyrin-based SOD mimics and peroxynitrite scavengers for pre-clinical mechanistic/therapeutic purposes. *J. Pharm. Biomed. Anal.* 48 1046–1049. 10.1016/j.jpba.2008.08.005 18804338 PMC2587431

[B45] Rong Y. Doctrow S. R. Tocco G. Baudry M. (1999). EUK-134, a synthetic superoxide dismutase and catalase mimetic, prevents oxidative stress and attenuates kainate-induced neuropathology. *Proc. Natl. Acad. Sci. U.S.A.* 96 9897–9902. 10.1073/pnas.96.17.9897 10449791 PMC22307

[B46] Rowley S. Patel M. (2013). Mitochondrial involvement and oxidative stress in temporal lobe epilepsy. *Free Radic. Biol. Med.* 62 121–131. 10.1016/j.freeradbiomed.2013.02.002 23411150 PMC4043127

[B47] Ryan K. Backos D. S. Reigan P. Patel M. (2012). Post-translational oxidative modification and inactivation of mitochondrial complex I in epileptogenesis. *J. Neurosci.* 32 11250–11258. 10.1523/JNEUROSCI.0907-12.2012 22895709 PMC3518304

[B48] Ryan K. Liang L. P. Rivard C. Patel M. (2014). Temporal and spatial increase of reactive nitrogen species in the kainate model of temporal lobe epilepsy. *Neurobiol. Dis.* 64 8–15. 10.1016/j.nbd.2013.12.006 24361554 PMC4872513

[B49] Sahebkar A. (2010). Neuroprotective effects of resveratrol: potential mechanisms. *Neurochem. Int.* 57 621–622. 10.1016/j.neuint.2010.06.014 20600434

[B50] Sakata Y. Zhuang H. Kwansa H. Koehler R. C. Dore S. (2010). Resveratrol protects against experimental stroke: putative neuroprotective role of heme oxygenase 1. *Exp. Neurol.* 224 325–329. 10.1016/j.expneurol.2010.03.032 20381489 PMC2885554

[B51] Sheng H. Chaparro R. E. Sasaki T. Izutsu M. Pearlstein R. D. Tovmasyan A. (2014). Metalloporphyrins as therapeutic catalytic oxidoreductants in central nervous system disorders. *Antioxid Redox Signal* 20 2437–2464. 10.1089/ars.2013.5413 23706004

[B52] Shetty A. K. (2011). Promise of resveratrol for easing status epilepticus and epilepsy. *Pharmacol. Ther.* 131 269–286. 10.1016/j.pharmthera.2011.04.008 21554899 PMC3133838

[B53] Shin E. J. Jeong J. H. Chung Y. H. Kim W. K. Ko K. H. Bach J. H. (2011). Role of oxidative stress in epileptic seizures. *Neurochem. Int.* 59 122–137. 10.1016/j.neuint.2011.03.025 21672578 PMC3606551

[B54] Sipos I. Tretter L. Adam-Vizi V. (2003). Quantitative relationship between inhibition of respiratory complexes and formation of reactive oxygen species in isolated nerve terminals. *J. Neurochem.* 84 112–118. 12485407 10.1046/j.1471-4159.2003.01513.x

[B55] Tomaciello F. Leclercq K. Kaminski R. M. (2016). Resveratrol lacks protective activity against acute seizures in mouse models. *Neurosci. Lett.* 632 199–203. 10.1016/j.neulet.2016.09.002 27600732

[B56] Waldbaum S. Patel M. (2010). Mitochondria, oxidative stress, and temporal lobe epilepsy. *Epilepsy Res.* 88 23–45. 10.1016/j.eplepsyres.2009.09.020 19850449 PMC3236664

[B57] Wang S. J. Bo Q. Y. Zhao X. H. Yang X. Chi Z. F. Liu X. W. (2013). Resveratrol pre-treatment reduces early inflammatory responses induced by status epilepticus via mTOR signaling. *Brain Res.* 1492 122–129. 10.1016/j.brainres.2012.11.027 23211629

[B58] Wang W. Wu Y. Zhang G. Fang H. Wang H. Zang H. (2014). Activation of Nrf2-ARE signal pathway protects the brain from damage induced by epileptic seizure. *Brain Res.* 1544 54–61. 10.1016/j.brainres.2013.12.004 24333359

[B59] Williams S. Hamil N. Abramov A. Y. Walker M. C. Kovac S. (2015). Status epilepticus results in persistent overproduction of reactive oxygen species, inhibition of which is neuroprotective. *Neuroscience* 303 160–165. 10.1016/j.neuroscience.2015.07.005 26162241

[B60] Wu Z. Xu Q. Zhang L. Kong D. Ma R. Wang L. (2009). Protective effect of resveratrol against kainate-induced temporal lobe epilepsy in rats. *Neurochem. Res.* 34 1393–1400. 10.1007/s11064-009-9920-0 19219549

[B61] Zielonka J. Kalyanaraman B. (2010). Hydroethidine- and MitoSOX-derived red fluorescence is not a reliable indicator of intracellular superoxide formation: another inconvenient truth. *Free Radic. Biol. Med.* 48 983–1001. 10.1016/j.freeradbiomed.2010.01.028 20116425 PMC3587154

